# Do potent immobilising-opioids induce different physiological effects in impala and blesbok?

**DOI:** 10.4102/jsava.v91i0.2038

**Published:** 2020-08-06

**Authors:** Silke Pfitzer, Michael Laurence, Liesel Laubscher, Jacobus P. Raath, Kristin Warren, Rebecca Vaughan-Higgins, Leith R.C. Meyer

**Affiliations:** 1 School of Veterinary Medicine, College of Science, Health, Engineering and Education, Murdoch University, Perth, Australia; 2 School of Biology and Environmental Sciences, Faculty of Agriculture and Natural Sciences, University of Mpumalanga, Nelspruit, South Africa; 3 Department of Animal Science, University of Stellenbosch, Cape Town, South Africa; 4 Wildlife Pharmaceuticals South Africa (Pty) Ltd, White River, South Africa; 5 Centre for Veterinary Wildlife Studies and Department of Paraclinical Sciences, Faculty of Veterinary Science, University of Pretoria, Pretoria, South Africa

**Keywords:** Blesbok, etorphine, immobilisation, impala, opioids, thiafentanil

## Abstract

Potent opioids are known to cause negative alterations to the physiology of immobilised antelope. How these effects differ between species has not been studied. This study aimed to compare time to recumbence and effects of opioid-based immobilisation on the physiology of impala (*Aepyceros melampus*) and blesbok (*Damaliscus pygargus phillipsi*). Eight animals of each species were immobilised, with 0.09 mg/kg etorphine and 0.09 mg/kg thiafentanil respectively, in a randomised two-way cross-over study. Variables measured and analysed by means of a linear mixed model included time to recumbence, heart rate, respiratory rate, arterial blood pressure, blood gases, lactate and glucose. In blesbok, mean time to recumbence was not significantly different with either drug (2.5 minutes and 2.2 min, respectively), but in impala thiafentanil achieved a shorter time to recumbence (2.0 min) than etorphine (3.9 min). Mean heart rates of immobilised impala were within reported physiological limits, but lower in immobilised blesbok when both opioids were used (35 beats/min to 44 beats/min vs. 104 ± 1.4 beats/min resting heart rate). Impala developed severe respiratory compromise and hypoxaemia from both opioids (overall mean PaO_2_ values ranged from 38 mmHg to 59 mmHg over 30 min). In contrast, blesbok developed only moderate compromise. Therefore, significantly different species-specific physiological responses to potent opioid drugs exist in blesbok and impala. Given that these different responses are clinically relevant, extrapolation of immobilising drug effects from one species of African ungulate to another is not recommended.

## Introduction

When immobilising drugs are developed for use in wild African ungulates, it is often difficult to make dose recommendations owing to the wide variety of species in this group and the lack of reported efficacy studies (Hernandez 2005). When investigating the physiological response to drugs in wild ungulates, domestic species are often used as models (Harthoorn 1967; Heard et al. 1996; Izwan et al. 2018; Meyer et al. 2015; Meyer, Fuller & Mitchell 2006; O’Dell et al. 2017; Pfitzer et al. 2019a). The domestic animal model approach is usually then followed by the selection of wild ungulate species thought to be representative of the domestic animal. The species most commonly used for this purpose is the impala. Impalas are easily kept in zoo as they are, not classified as threatened and are readily available in the wild and in intensive farming operations in Southern Africa (Harthoorn 1967; Janssen et al. 1993; Meyer et al. 2008; Perrin et al. 2015; Pfitzer et al. 2019b; Zeiler & Meyer 2017a, 2017b). Looking at the phylogenetic tree of the subfamily of Antilopinae, impala are somewhat removed from other subfamilies commonly captured by immobilisation such as Hippotraginae, which include sable antelope (*Hippotragus niger*), roan antelope (*Hippotragus equinus*) and oryx (*Oryx gazella*); and Alcelaphinae, which include blesbok, two wildebeest species (*Connochaetes* spp.), hartebeest (*Alcelaphus* spp.) and tsessebe (*Damaliscus* spp.) (Chen et al. 2019; Hernandez Fernandez & Vrba 2005). Impala are considered a difficult species to immobilise (Perrin et al. 2015; Pfitzer et al. 2019b; Zeiler & Meyer 2017a, 2017b). Their flighty nature and fine body structure can lead to physical injury when they are darted. Furthermore, their physiological response to opioids seems unpredictable and frequent deaths have been reported (Meyer et al. 2010; Perrin et al. 2015; Zeiler & Meyer 2017a, 2017b). Accordingly, the physiological response of impala to opioids might not necessarily be representative and comparable to many other antelope species. In contrast, blesbok seem to have a more predictable response to opioids and are considered an easy species to immobilise (Pfitzer et al. 2019b). Blesbok, belonging to the subfamily Alcelaphinae, are phylogenetically more closely related to many African antelope species (Chen et al. 2019; Hernandez Fernandez & Vrba 2005). Blesbok were chosen for this study because, like impala, blesbok are seasonal breeders, are readily available in Southern Africa and are not threatened (Furstenburg 2016a, 2016b). Blesbok also live in a herd and can be housed in captivity in groups. As both sexes have horns, long-term confinement can be a challenge as fighting and injuries occur (Pfitzer et al. 2019b). These are some of the reasons why blesbok are not commonly kept in zoo collections and have not been commonly used for ex-situ wildlife research. Only recently have several capture-related research projects been carried out using blesbok (Du Plessis 2018; Fitte 2017; Sawicka et al. 2014).

The aim of this experiment was to compare the physiological responses of impala and blesbok to two potent opioids which are commonly used to immobilise wild antelope species. It was hypothesised that the opioids will affect each species differently and that those effects are of clinical relevance.

## Materials and methods

This study was part of a larger study evaluating the effects of etorphine and thiafentanil in blesbok and impala respectively.

Eight wild-captured female impala weighing 37 kg (standard error [SE] 2) and eight wild-captured female blesbok weighing 58 kg (SE 1.45) were selected for this experiment and held at the Wildlife Pharmaceuticals Wildlife Research Facility, Mpumalanga, South Africa (25°31’25.2” S, 31°06’50.8” E). The research animals were all adult females of similar size and in good health in order to minimise variability that could confound the results. Horns of the blesbok were piped with plastic pipes to prevent horn injuries commonly encountered in captive blesbok.

The wildlife enclosures were constructed according to national guidelines and consisted of several compartments (SABS 2004). The animals were held separated by species and in groups of four. After an initial adjustment period of 2 weeks post-delivery, the animals were immobilised, marked, weighed and subjected to a veterinary health examination one week before the trial.

The immobilising drugs etorphine (Captivon, 9.8 mg/mL, Wildlife Pharmaceuticals [Pty] Ltd, South Africa) and thiafentanil (Thianil, 10 mg/mL, Wildlife Pharmaceuticals [Pty] Ltd, South Africa) were administered intramuscularly via remote injection by darting into the gluteus muscles. Darting equipment consisted of a gas-powered dart projector (X-Caliber; Pneu-Dart Inc., Pennsylvania, United States [US]) combined with 1 mL P-type Pneu-Darts with 1.9 cm barbed needles (Pneu-Dart Inc., Pennsylvania, US). Animals were darted on two separate occasions with a wash-out period of two weeks between each occasion. Each antelope received both drug treatments which were allocated to animals at random in a two-way cross-over design. One treatment consisted of 0.09 mg/kg etorphine and the other treatment consisted of 0.09 mg/kg thiafentanil. The immobilisation efficacy of these doses had been determined in a previous study (Pfitzer et al. 2019b).

As soon as an animal became recumbent and could be approached, it was blindfolded and cotton wool was inserted into its ears to minimise external stimuli. The animal was then placed onto a stretcher in sternal recumbency with its head held up and nose pointing downwards to ensure patency of the upper airway. It was carried to the monitoring table and kept in this position throughout the monitoring period.

Time to recumbence was measured by means of a stopwatch function on a wristwatch (G-Shock, Casio Computer Co., Ltd., Japan) and defined as time from dart injection to when the animal was recumbent and unable to rise.

Monitoring and recording began at 5 min after an animal became recumbent and was continued every 5 min until 40 min post-recumbence. Rectal body temperature was measured in °C by means of a modified handheld digital thermometer (Hanna Checktemp 1, Hanna Instruments [Pty] Ltd, Nebraska, US). The environmental temperature was measured by the Weather^+^ Bluetooth Sensor (Oregon Scientific, Oregon, US). Barometric pressure was measured by the epoc portable blood gas analyser (epoc Blood Analysis System, Epocal, Ontario, Canada). This instrument was also used to analyse arterial blood pH, glucose in mmol/L, lactate in mmol/L, arterial partial pressure of carbon dioxide (PaCO_2_) in mmHg at 37 °C and arterial partial pressure of oxygen (PaO_2_) in mmHg at 37 °C. The respiratory rate (breaths/minute) was measured manually by means of visual observation of chest expansions and auscultation with a stethoscope for 1 min (Littmann Classic II, 3M^TM^, Minnesota, US). Heart rate (beats/minute) was also measured with a stethoscope in the same manner as respiratory rate. Systolic arterial pressure (SAP) in mmHg, diastolic arterial pressure (DAP) in mmHg and mean arterial pressure (MAP) in mmHg were determined by means of an intra-arterial blood pressure measurement at the auricular (*Arteria auricularis*) or pedal arteries (*A. digitalis*). A Deltran II pressure transducer (Utah Medical, Utah, US) connected to an IntraTorr blood pressure monitor (IntraTorr, IntraVitals, United Kingdom [UK]) were used for this purpose. The transducer was placed near the scapulohumeral joint (heart base) of the animal. Arterial blood was drawn anaerobically in a heparinised blood gas syringe (BD A-Line, Becton, Dickinson and Company, UK) and subjected to blood gas analysis within 5 min using the epoc portable blood gas analyser with epoc BGEM test cards (BGEM Smart Cards, Epocal, Ontario, Canada). Samples were collected at the frequencies of 5, 10, 15, 20 and 30 min after recumbence. The alveolar-arterial oxygen (A-a) gradient was calculated according to the publication by Meyer et al. (2010). The A-a gradient was calculated for an open system (constant pressure) from the formula:

$${\text{FiO}}_{2}(\text{Pb}-{\text{PH}}_{2}\text{O})-{\text{PaCO}}_{2}-{\text{PaO}}_{2},$$ [Eqn 1]

where FiO_2_ is the fractional inspired oxygen (0.209), Pb is the measured barometric pressure (mmHg) and PH_2_O is the water vapour pressure of saturated air in the alveoli. PH_2_O (mmHg) was calculated as 4.58 e{(17.27 Tb)/(237.3 + Tb)}, where Tb is the body temperature. The researchers assumed that the partial pressure of CO_2_ in the alveoli was equal to the arterial partial pressure of CO_2_. A mean PaO_2_ < 80 mmHg was defined as moderate hypoxaemia whilst a mean PaO_2_ of < 60 mmHg was defined as severe hypoxaemia.

After the 40-min monitoring period was over, the immobilisation of each animal was reversed with intravenous naltrexone (Trexonil, 50 mg/mL, Wildlife Pharmaceuticals [Pty] Ltd, South Africa), injected at a ratio of 20 mg naltrexone to 1 mg etorphine and 10 mg naltrexone per 1 mg thiafentanil.

The partial reversal agent butorphanol (Butonil 50 mg/mL, Wildlife Pharmaceuticals [Pty] Ltd, South Africa) as well as naltrexone were kept on hand to partially reverse or fully reverse the immobilisation in case of adverse anaesthetic events, such as potentially life-threatening apnoea of a duration exceeding 1.5 min. No animals needed this treatment.

Ambient temperatures during the blesbok experiments with etorphine were 24.9 ºC (SE 4.99) and during thiafentanil treatment they were 24.3 ºC (SE 1.55). The mean barometric pressure was 698 (SE 1.80) mmHg.

For the impala, ambient temperatures measured during the etorphine treatment were 20.7 ºC (SE 4.52) and during thiafentanil treatment they were 21.9 ºC (SE 4.68). The mean barometric pressure was 697 (SE 2.34) mmHg.

### Data analysis

Analyses were performed with Genstat Version 17 (VSN International, UK) to determine whether or not there was a species-specific difference between the two opioids on the physiological variables of interest. A linear mixed model was fitted to the monitored physiological variables and immobilisation score. Each fixed model included the following factors: species-specific effects, treatment effects, species-specific by treatment interactions, the effects of time after recumbence, and interactions between time after recumbence and species and treatment. The random model included terms for animal, animal by treatment and animal by treatment by minutes after recumbence. The residual variance or covariance model included correlations between measurements made on the same animal for a specific treatment and different variances for each animal.

Time to recumbence was analysed using a linear mixed model which included species-specific effects, treatment effects and their interaction in the fixed model, and animal by treatment effects in the random model. The residual variance model included different variances for each species.

Non-significant random effects and covariances were removed from the model before the significance of fixed effects was assessed. Post-analysis residual plots were examined to confirm that the assumptions required for a linear model were met. When interpreting results, only effects that included a species-specific interaction with treatment were of interest as these indicated differences in the species’ response to treatment. A *p*-value of less than 0.05 was considered significant. Post hoc comparisons were made for significant species-specific interactions using a 5% least significant difference (5% LSD). In figures, the 5% LSD is used to illustrate whether or not differences between the species means are significant. Any difference larger than the 5% LSD is considered a significant result. Results are presented as mean and standard error (SE).

### Ethical considerations

Ethical approval to conduct the study was obtained from the Murdoch University, ethical clearance number R 3039/18.

## Results

Mean rectal temperatures of blesbok during the monitoring period varied between 39.0 ºC and 39.2 ºC (SE 0.08) with the etorphine treatment, and between 38.9 ºC and 39.0 ºC (SE 0.08) with the thiafentanil treatment. In impala, mean rectal temperatures during etorphine immobilisation varied from 38.7 ºC to 39.1 ºC (SE 0.12) and with the thiafentanil treatment rectal temperatures varied from 38.8 ºC to 39.2 ºC (SE 0.12).

There was a significant species by treatment interaction for time to recumbence (*p* = 0.009). In blesbok, there were no significant differences between treatments for mean time to recumbence. The mean time to recumbence in blesbok immobilised with etorphine was 2.5 min (SE 0.14) and with thiafentanil, 2.2 min (SE 0.14). This finding was in contrast to impala, where the mean time to recumbence was faster (*p* = 0.007), with the thiafentanil treatment at 2.0 min (SE 0.17) compared to etorphine treatment at 3.9 min (SE 0.19).

**FIGURE 1:**
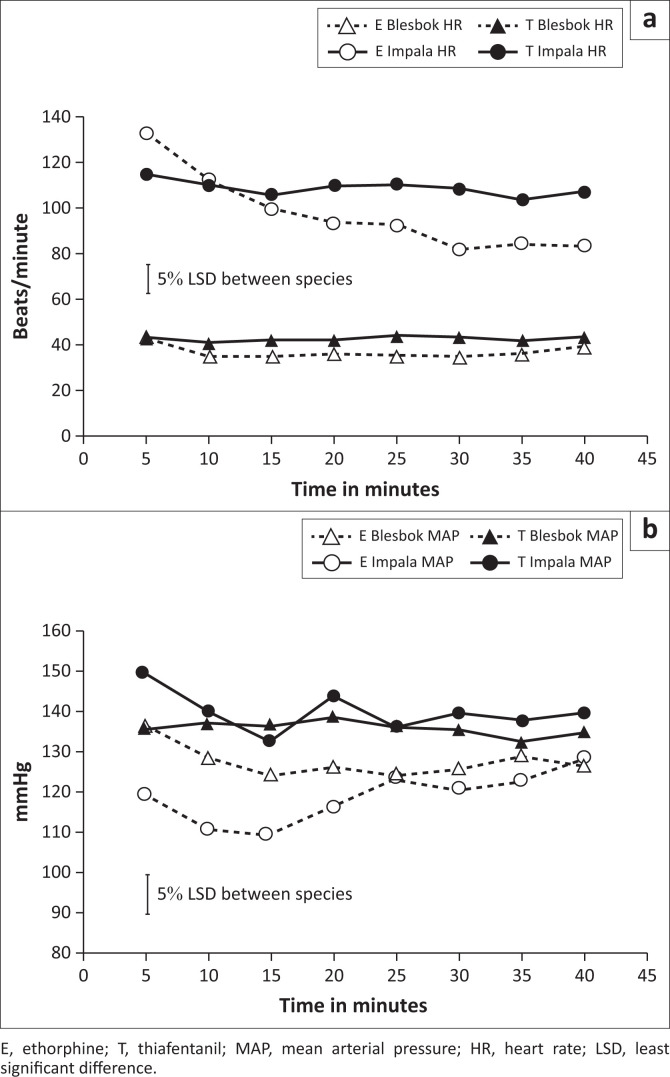
Heart rate (HR) in beats/minute (a) and mean arterial pressure (MAP) in mmHg (b) over time (minutes) of blesbok (*Damaliscus pygargus phillipsi*) and impala (*Aepyceros melampus*) immobilised with 0.09 mg/kg etorphine (E) or thiafentanil (T) respectively. Time was measured from when an animal became recumbent as a result of the immobilisation.

There was a significant interaction of species, treatment and time (*p* = 0.007) for heart rate. Blesbok, when treated with either opioid, developed much lower overall mean heart rates (etorphine mean = 37; thiafentanil mean = 43 beats/min [SE 1.4]) compared to impala etorphine mean = 98; thiafentanil mean = 109 beats/min [SE 4.0]). The heart rate of impala, when treated with etorphine, started with a mean of 133 beats/min at 5 min but dropped within 30 min to 82 beats/min (SE 4.5). The range of heart rate actually measured in impala during these experiments varied from 52 beats/min to 190 beats/min. This considerable change over time and variability was not present in blesbok (Figure 1a).

There was a significant species by treatment by time interaction for mean arterial pressure (MAP) (*p* = 0.007).

Mean arterial pressure was elevated in both species with either drug throughout the monitoring period but more so when thiafentanil was administered (*p* < 0.001). In blesbok, the mean MAP was 128 mmHg and 136 mmHg (SE 2.4) with etorphine and thiafentanil respectively. In impala, the mean MAP was 119 mmHg with etorphine and 140 mmHg (SE 2.2) with thiafentanil (Figure 1b).

**FIGURE 2:**
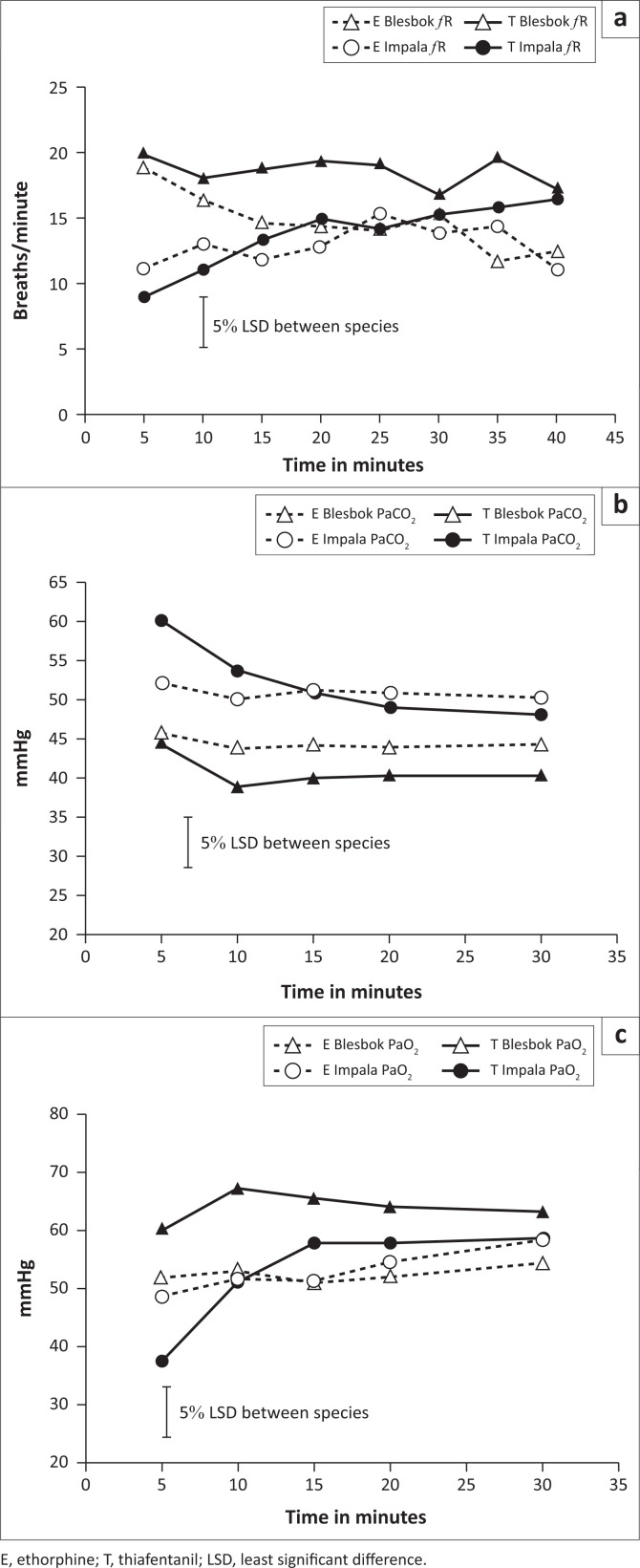
Respiratory rate in breaths/minute (a), PaCO_2_ (b) and PaO_2_ (c) in mmHg over time (minutes) of blesbok (*Damaliscus pygargus phillipsi*) and impala (*Aepyceros melampus*) immobilised with 0.09 mg/kg etorphine (E) or thiafentanil (T) respectively. Time was measured from when an animal became recumbent as a result of the immobilisation.

There was a species by treatment interaction (*p* = 0.036) as well as a species by time interaction (*p* < 0.001) for respiratory rate. Blesbok immobilised with thiafentanil over the entire monitoring period showed the highest respiratory rate. Impala, when immobilised with either opioid, showed very low respiratory rates especially in the first 15 min (Figure 2a). Twenty per cent of impala suffered from initial induction apnoea when immobilised with thiafentanil. Induction apnoea was defined as no breath taken for over a minute. The apnoea corrected itself within this time frame and the animals were just monitored and stimulated by pulling the tongue or pinching the nasal septum.

There was a species by treatment by time interaction for PaCO_2_ (*p* = 0.027). For the mean PaCO_2_ a significant species difference could also be observed as PaCO_2_ values were higher in impala compared to blesbok with both treatments (*p* = 0.002) (Figure 2a).

There was a significant species by treatment interaction for PaO_2_ (*p* = 0.006). Thiafentanil treatment of impala in the beginning elicited an extremely low PaO_2_ of 38 mmHg, which then improved within the first 10 min and reached 59 mmHg (SE 1.7) after 30 min. Blesbok, in contrast, experienced milder hypoxaemia with thiafentanil throughout the monitoring period with mean PaO_2_ values ranging from 61 mmHg to 68 mmHg (SE 1.5) (Figure 2b).

**FIGURE 3:**
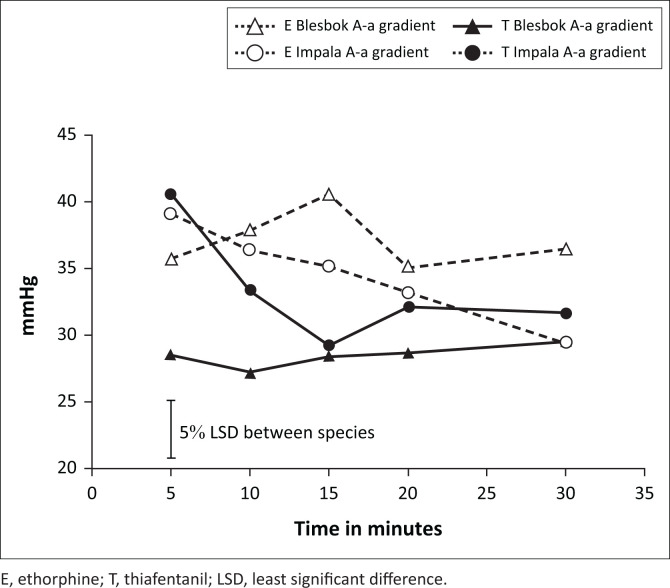
A-a gradient in mmHg over time (minutes) of blesbok (*Damaliscus pygargus phillipsi*) and impala (*Aepyceros melampus*) immobilised with 0.09 mg/kg etorphine (E) or thiafentanil (T) respectively. Time was measured from when an animal became recumbent as a result of the immobilisation.

There was a species by treatment interaction (*p* = 0.009) for the A-a gradient. In blesbok, the A-a gradient was significantly lower when animals were treated with thiafentanil (mean = 28 mmHg [SE 1.0]) compared to etorphine (mean = 37 mmHg [SE 1.0]). This difference between the two opioids was not observed in impala in which the mean A-a gradient measured 34 mmHg and 33 mmHg (SE 0.7) for etorphine and thiafentanil respectively (Figure 3).

There was significant species by treatment interaction for pH (*p* < 0.001). With either opioid treatment, blesbok had higher arterial pH values compared to impala (Table 1a).

**TABLE 1a:** Species comparison of arterial blood pH, lactate in mmol/L and glucose in mmol/L over time and standard error during immobilisation, with etorphine 0.09 mg/kg and thiafentanil 0.09 mg/kg, of blesbok (*Damaliscus pygargus phillipsi*) and impala (*Aepyceros melampus*) respectively.

Variable	Species	Time after recumbence with etorphine in minutes					SE
		5	10	15	20	30	
pH	Blesbok	7.42	7.43	7.43	7.44	7.44	0.005
	Impala	7.30	7.32	7.34	7.36	7.39	0.007
Lactate	Blesbok	1.4	1.1	1.0	0.9	0.7	0.22
	Impala	5.2	4.2	3.2	2.5	1.5	0.26
Glucose	Blesbok	5.3	5.2	5.1	5.0	5.1	0.11
	Impala	7.7	7.6	7.3	7.0	6.4	0.31

Note: Time was measured from when an animal became recumbent as a result of the immobilisation.

SE, standard error.

There was a significant species by time interaction (*p* = 0.002) for lactate concentrations. Impala developed higher lactate values than blesbok, irrespective of the treatment. In both species, lactate values decreased over time (Table 1a).

There was a significant species difference in glucose concentrations (*p* < 0.001); impala developed higher glucose values than blesbok for both opioid treatments. Glucose decreased over time in impala with both treatments, but glucose values did not decrease in blesbok (Table 1b).

**TABLE 1b:** Species comparison of arterial blood pH, lactate in mmol/L and glucose in mmol/L over time and standard error during immobilisation, with etorphine 0.09 mg/kg and thiafentanil 0.09 mg/kg, of blesbok (*Damaliscus pygargus phillipsi*) and impala (*Aepyceros melampus*) respectively.

Variable	Species	Time after recumbence with thiafentanil in minutes					SE
		5	10	15	20	30	
pH	Blesbok	7.44	7.47	7.47	7.48	7.49	0.005
	Impala	7.27	7.31	7.33	7.35	7.40	0.007
Lactate	Blesbok	1.2	0.9	0.8	0.7	0.5	0.22
	Impala	5.3	4.4	3.5	2.7	1.7	0.26
Glucose	Blesbok	4.5	4.3	4.2	4.2	4.5	0.11
	Impala	8.8	9.0	8.8	8.6	8.1	0.31

Note: Time was measured from when an animal became recumbent as a result of the immobilisation.

SE, standard error.

## Discussion

Comparing the physiological response to immobilisation between two common antelope species yielded interesting findings. In general, it is apparent that different species respond differently to commonly administered drugs. Specifically, it appeared that blesbok were less subject to respiratory depression. These findings have implications for the use of immobilising agents in the field and in different species.

Both species of antelope were successfully immobilised for 40 min with both opioids, using doses of nearly double the dose used when either opioid is combined with a tranquilliser or sedative (Kock & Burroughs 2012).

Time to recumbence is an important factor under field conditions. Shorter time to recumbence reduces exposure to stressors and facilitates faster retrieval of the animal. A short time to recumbence also lessens the risk of animals disappearing or being attacked by herd members or predators whilst the drugs are taking effect. Therefore, the significantly shorter time to recumbence measured in impala when immobilised with thiafentanil in this experiment is of great clinical relevance. However, this factor must be weighed up against the severe adverse cardiopulmonary effects that this drug induces when a clinician decides to use it in the field. Importantly, this shorter time to recumbence does not seem to be ubiquitous in antelope as there was no difference in this variable when the two opioids were compared in the blesbok.

A further important difference, from a clinical perspective, between the two species was the heart rate. In blesbok treated with opioids, the heart rate was much slower (means measured between 36 beats/min and 45 beats/min) than the mean heart rate that was measured in awake blesbok at rest (104 beats/min) (Du Plessis 2008). This could be interpreted as classic reflex bradycardia as a result of opioid-induced vasoconstriction and hypertension. Resting heart rates in impala are not known. However, in sheep and goats, which are similar in size to impala, resting heart rate is 70–110 beats/min (Izwan et al. 2018; Meyer et al. 2015; Sjaastad, Sand & Hoove 2016). In contrast to blesbok, impala during the first 10 min developed a higher heart rate which showed wide variation between individuals and also over time (Figure 1a). Species differences in domestic and wild ungulates with regard to the effects of opioids on the heart rate have been reported previously (Harthoorn 1967; Izwan et al. 2018). In a comparative experiment between sheep and goats, sheep developed a significantly elevated heart rate with etorphine treatment, whilst the same treatment did not affect the heart rate of goats. At the same time, the stroke volume in sheep decreased and therefore the elevated heart rate did not result in changes in cardiac output (Izwan et al. 2018).

Despite the species difference in heart rate, both species developed an elevated MAP with both opioids. This elevation was more severe when thiafentanil was used, with mean MAP values of 136 mmHg and 140 mmHg for blesbok and impala respectively (Figure 1b). The MAP of most healthy ruminants should measure around 115 mmHg, irrespective of body size (Prothero 2015). In a field setting, heart rate and blood pressure are important variables as they may influence the choice of sedatives or tranquillisers to be combined with an opioid for immobilisation. For example, it is common practice amongst wildlife veterinarians to combine etorphine with the butyrophenone derivative azaperone as it is a tranquilliser but also causes vasodilation and has anti-hypertensive effects (Kock & Burroughs 2012). This study highlights the fact that opioids have profound cardiovascular effects that need to be studied in more detail to understand their full physiological effects, reveal their species-specific risks and understand how additive drugs like sedatives and tranquillisers alter these.

Du Plessis (2018) reported a mean respiratory rate of recumbent resting blesbok of 13 breaths/min, and for standing blesbok a rate of 20 breaths/min. The respiratory rate of impala at rest has been reported as 20.0 ± 8 breaths/min (Cheney & Hattingh 1987). Blood gas values for sheep and goats at rest were measured by Ismail, Jawasreh and Al-Majali (2010) who reported a mean PaCO_2_ of 41 ± 2.5 for sheep and 40 ± 7 for goats. Impala, especially at the start of the monitoring period, displayed a low respiratory rate and hypercapnia (PaCO_2_ means = 51 mmHg [etorphine] and 52 mmHg [thiafentanil]) with both opioid treatments. Impala also suffered from severe hypoxaemia (PaO_2_ means = 53 mmHg [both drugs]).

PaCO_2_ values of blesbok (means = 45 mmHg [etorphine] and 41 mmHg [thiafentanil]) were within the upper physiological range of conscious small ruminants (Ismail et al. 2010), indicating adequate ventilation during immobilisation. Despite adequate ventilation, blesbok suffered from moderate hypoxaemia with thiafentanil (mean PaO_2_ 61 mmHg to 68 mmHg) and severe hypoxaemia with the etorphine treatment (mean PaO_2_ 51 mmHg to 54 mmHg) (Figure 2b, c). Possible reasons for hypoxaemia in this case can be explained by looking at the A-a gradient, which was elevated. In the literature, A-a gradients for small ruminants at rest were reported between 20 mmHg and 25 mmHg (Izwan et al. 2018; Meyer et al. 2006). Therefore, the mean A-a gradients of the blesbok as well as the impala treated with etorphine should be considered elevated as they measured 37 and 35 mmHg respectively. This finding indicates that etorphine caused an equally elevated A-a gradient in both species. Thiafentanil, in contrast, caused a more severe A-a gradient elevation in impala (mean = 33 mmHg) compared to blesbok (mean = 28 mmHg). A ventilation-perfusion mismatch or right-left shunting of lung portions might have been the cause for inadequate oxygen diffusion indicated by the elevated A-a gradients (Fahlman et al. 2016; Meyer et al. 2015). Opioid-induced pulmonary vasoconstriction, resulting in pulmonary hypertension, might have also led to inadequate oxygen diffusion as a result of pulmonary congestion and/or oedema. Vasoconstriction could have also caused a faster blood flow which might have hindered gas exchange, as capillary blood might have passed the alveoli too fast to enable effective oxygen diffusion (Hattingh et al. 1994; Meyer et al. 2015). Furthermore, opioid-induced hypermetabolism (Buss et al. 2018) may have increased systemic oxygen extraction which possibly could have also increased the A-a gradient.

Clearly, if the respiratory rate was the only assessment of respiration, then hypoxaemia in blesbok under etorphine immobilisation would have been overlooked. The measurement of blood gases, although expensive and not always practical to use in the field, should therefore not be underestimated as an important monitoring tool.

Initially elevated lactate in the impala could have been associated with an initial exaggerated stress response of these animals to the capture procedure. A stress response would lead to an increased metabolism and therefore increase the oxygen demand, further compounding the hypoxaemia (Buss et al. 2018). The assumption that impala not only suffered from hypoxaemia due to opioid-induced respiratory compromise but also experienced a stress response is supported by the elevated glucose and an initially raised heart rate (Hattingh 1988). Blesbok, in contrast, did not show signs of such a severe initial stress response.

## Implications

Impala seem to be more fractious in nature compared to blesbok and a stress response, coupled with a greater sensitivity to opioid-induced respiratory compromise, may explain why impala are more at risk and considered to be one of the more challenging species to immobilise. Not only are they a small and fragile target to dart but the severe respiratory disturbances, coupled with stress-related physiological derangements, can also be fatal if not immediately addressed. Furthermore, the time to recumbence induced by thiafentanil and etorphine differed in impala, and this should be taken into consideration when the appropriate opioid is chosen for the capture of this species.

In contrast, there was no difference in time to recumbence between the opioids in blesbok. Also, in blesbok, a heart rate lower than the resting heart rate can be expected when animals are immobilised with these opioids. This lower heart rate is likely a baroreceptor reflex to opioid-induced vasoconstriction and hypertension. Heart rate, respiratory rate and blood gas variables did not differ as much over the monitoring time in blesbok as was observed in impala. Furthermore, hypoxaemia and hypercapnia in the immobilised blesbok were not as severe as in impala.

Impala, due to their availability and presence in many zoos, have been commonly studied to understand the physiological effects of immobilising drugs. However, as this study indicates, the response to immobilising drugs is not uniform across all antelope species. Therefore, the practice of only using impala for such studies may result in misleading perceptions. Standardised monitoring and data analysis, from multiple antelope species immobilised during routine management procedures, should be undertaken in the future in order to better understand the physiological effects of the drugs used, and enable more clinically relevant, species-specific recommendations for immobilisation.

## Study limitations

The small sample size is acknowledged as a limitation of this study. However, due to logistics, conservation status and ethical concerns, experiments in wildlife are often carried out with small numbers of 6 to 12 animals (Meyer et al. 2018; Pfitzer et al. 2019b; Zeiler & Meyer 2017a). Only healthy, adult animals which were adapted to captivity were part of this experiment. In a field situation, drug doses might have to be adjusted according to the physiological status of the animals, such as size, age, nutritional status and pregnancy. For logistical and animal welfare reasons, only females were used in this study. Females may have slightly different drug and stress-related responses compared to males; however, these differences need to be confirmed.

## Conclusion

This experiment confirms the initial hypothesis that significantly different, species-specific physiological responses to opioid immobilising drugs exist between blesbok and impala. The most profound difference was that blesbok seem less affected by respiratory compromise than impala. Impala developed severe hypoxaemia, which is not only due to opioid-induced hypoventilation but also likely due to the impairment of oxygen diffusion as well as hypermetabolism. Some of the changes observed in impala might also be strongly associated with a species-specific stress response to capture, rather than just the actions of the opioids themselves. Extrapolation of immobilisation results from one species of African ungulate to another is not recommended, given the significant differences in the way that these two species responded to the immobilisation.
